# Magnetic Anomaly Detection Based on a Compound Tri-Stable Stochastic Resonance System

**DOI:** 10.3390/s23229293

**Published:** 2023-11-20

**Authors:** Jinbo Huang, Zhen Zheng, Yu Zhou, Yuran Tan, Chengjun Wang, Guangbo Xu, Bingting Zha

**Affiliations:** 1 School of Mechanical Engineering, Nanjing University of Science and Technology, Nanjing 210094, China; huangjinbo@njust.edu.cn (J.H.); zhengzhen@njust.edu.cn (Z.Z.); zy2020@njust.edu.cn (Y.Z.); tanyr@njust.edu.cn (Y.T.); wangchengjun@njust.edu.cn (C.W.); xgbxgb@njust.edu.cn (G.X.); 2 China and the Science and Technology on Electromechanical Dynamic Control Laboratory, Xi’an 710065, China

**Keywords:** magnetic anomaly detection, stochastic resonance, compound tri-stable, signal-to-noise ratio

## Abstract

In the case of strong background noise, a tri-stable stochastic resonance model has higher noise utilization than a bi-stable stochastic resonance (BSR) model for weak signal detection. However, the problem of severe system parameter coupling in a conventional tri-stable stochastic resonance model leads to difficulty in potential function regulation. In this paper, a new compound tri-stable stochastic resonance (CTSR) model is proposed to address this problem by combining a Gaussian Potential model and the mixed bi-stable model. The weak magnetic anomaly signal detection system consists of the CTSR system and judgment system based on statistical analysis. The system parameters are adjusted by using a quantum genetic algorithm (QGA) to optimize the output signal-to-noise ratio (SNR). The experimental results show that the CTSR system performs better than the traditional tri-stable stochastic resonance (TTSR) system and BSR system. When the input SNR is -8 dB, the detection probability of the CTSR system approaches 80%. Moreover, this detection system not only detects the magnetic anomaly signal but also retains information on the relative motion (heading) of the ferromagnetic target and the magnetic detection device.

## 1. Introduction

Magnetic anomaly detection (MAD) is a widely used passive magnetic target detection method. Its applications include surface ship target detection, underwater motion target monitoring, land target detection, and metal mine seismic activity identification [1,2,3,4,5]. MAD methods can be divided into two categories. The first is target-based detection methods, mainly typical of magnetic search systems, which are based on an assumption that the magnetometer will move relative to a target and are suitable for cases where the target motion obeys a specific tracking time pattern. The second is noise-based detection methods [6]. These methods are based on the statistical analysis of magnetometer noise and are suitable for cases where a mutual target and magnetometer cannot be assumed [7].

A stochastic resonance (SR) algorithm has two advantages in terms of implementation and detection performance. The stochastic resonance phenomenon was first discovered and proposed by R. Benzi et al. in their study of periodic recurrence in ancient meteorology [8]. The concept of stochastic resonance was extended to the broad category of “noise enhanced performance” [7]. Research on SR has focused on the fields of meteorology, living body neurology, and physics [9,10,11]. SR techniques have been widely used in the extraction and detection of weak signals [12]. Z. Qiao et al. introduced three types of asymmetric bistatic stochastic resonances by varying the depth and width of a typical bistatic stochastic resonance model of a double well potential and the depth and width of the left well. This method solves the effect of multiplicative and additive noise on the potential SR asymmetry and improves the output signal-to-noise ratio (SNR) [13]. In recent years, tri-stable systems have received increasing attention, and many different tri-stable system models have been proposed from different perspectives. H.B. Zhang et al. proposed a tri-stable stochastic resonance model. The model has a greater degree of noise energy transfer to the signal, and the output signal is significantly enhanced compared to a BSR system [14]. S.L. Lu et al. proposed a tri-stable stochastic resonance method to enrich the potential function model and proved that it performs better than conventional monostable stochastic resonance and BSR [15]. G. Zhang et al. proposed a piecewise unsaturated under-damped tri-stable stochastic resonance system to overcome the output saturation problem of classical tri-stable stochastic resonance systems [16].

It is essential to study MAD data based on the geomagnetic background [6]. In many cases, the detection range is limited by external magnetic noise rather than by the sensitivity of the sensor [17]. One of the main sources of magnetic noise is geomagnetic noise, whose power spectral density is 1/*f* ^α^(0 < α < 2) [18]. C.B. Wan et al. used a BSR method to detect magnetic anomalies, which can more effectively detect weak magnetic anomalies hidden in the noise background [19]. W. Liu et al. proposed a parallel monostable stochastic resonance system and searched for the optimal system parameters in the SR system to achieve good detection performance under different waveforms. The system improves the monostable stochastic resonance system, whose output is influenced by the peak signal and trough signal [20]. Z.Y. Liu et al. proposed an adaptive stochastic resonance system employing the kurtosis index as the criteria to automatically adjust the system structural parameters, which performs well in the detection of magnetic anomaly signals with background noise [21]. C.B. Wan et al. proposed a novel detection method based on the parallel stochastic resonance (PSR) system, which improves the SNR using the PSR system. The system performs better than the detector of a single stochastic resonance system [22]. H.X. Sun et al. proposed an adaptive cascade weak magnetic anomaly detection method based on the marine predator algorithm-stochastic resonance (MPA-SR) to solve the problem of the effective detection of weak magnetic anomaly signals in complex underwater environments. This detection method includes a cascade detection method with low-pass filtering, stochastic resonance, and threshold detection, which improves the detection probability of magnetic anomaly signals [23].

However, the traditional stochastic resonance encounters challenges in achieving a balance between constraint and continuity. Additionally, it poses difficulties in independently adjusting the depth and width of the potential well. Consequently, leveraging the effects of stochastic resonance at lower SNRs becomes challenging. In summary, a new CTSR model is proposed to further improve the SNR of magnetic anomaly detection and to preserve as many signal characteristics as possible. First, the generation of a magnetic anomaly signal is introduced. Second, this paper proposes the CTSR detection principle, designs the statistical filtering-based judgment system for magnetic anomaly signal detection, and optimizes the system parameters using a particle genetic algorithm. Finally, we conduct simulation tests to analyze the performance of the composite tri-stable stochastic resonance detector under different magnetic anomaly signals.

## 2. Related Work

### 2.1. Magnetic Anomaly Signal

**Figure 1:**
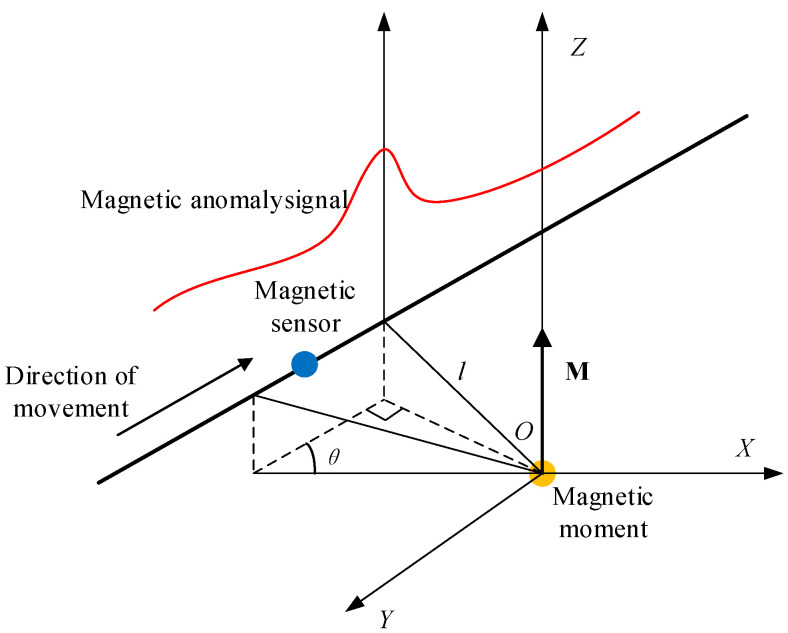
Magnetic anomaly detection model.

In the magnetic anomaly detection model (Figure 1), a magnetic target with the moment of **M** is located at the origin O of a Cartesian coordinate system, with **M** lying along the negative direction of the *Z*-axis. A sensor moves along a straight line with a relative angle *θ* between the search path and the positive *X*-axis, *l* is the closest contact distance between the detection path and the magnetic target, and *v* represents the sensor moving speed. The total magnetic field consists of the background magnetic field and the anomalous field generated by the ferromagnetic object, namely

(1) $$B_{t}=B_{e}+B_{r}$$

where **B***_t_* is the total magnetic field vector, **B***_e_* is the background magnetic field vector, and **B***_r_* is the magnetic anomaly field vector. In fact, $\left|B_{t}\right|\gg \left|B_{r}\right|$, so the magnetic distortion field can be expressed as

(2) $$\left|B_{r}\right|=\Delta T=\left|B_{t}\right|-\left|B_{e}\right|\approx \frac{\langle B_{e},B_{r}\rangle }{\left|B_{e}\right|}$$

where |**B***_t_*| is the total magnetic field scalar value, |**B***_e_*| is the background magnetic field scalar value, and |**B***_r_*| is the scalar value of the magnetic anomaly field vector.

**Figure 2:**
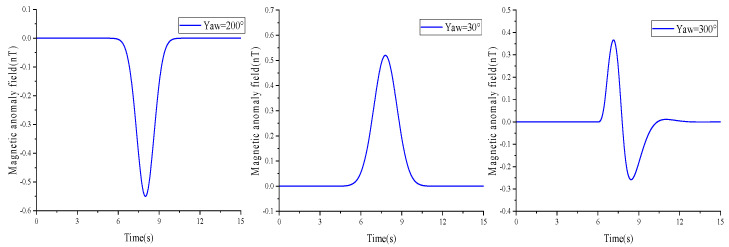
Simulation of a typical magnetic anomaly signal diagram.

The equivalent magnetic moment is set to **M** = (0, 0, 0.3)A∙m^2^, the magnetic sensor moves along the *θ* = 30°, 200°, and 300° directions, the speed is set to *v* = 5 m/s, the closest contact distance between the detection path and the magnetic target is *l* = 5 m, and the sampling frequency is 2 kHz. Combining Equations (1) and (2) and the simulation conditions, the simulation obtains three typical magnetic anomaly signals, as shown in Figure 2.

**Figure 3:**
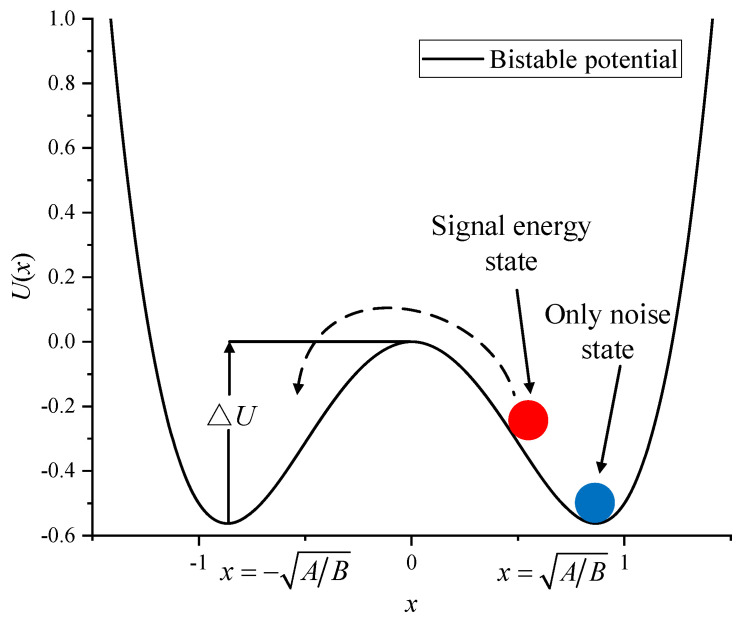
Stochastic resonance process.

When the input signal is just noise, the particle cannot cross the energy potential barrier into another potential well, as shown by the blue particle in Figure 3. When the input signal has a target signal, additive noise, and a well-coordinated nonlinear system, the particles remain in one potential well and may then cross the energy barrier into another potential well, as shown by the red particle in Figure 3. This phenomenon can be regarded as stochastic resonance.

### 2.2. Stochastic Resonance Principle

The traditional BSR model is expressed as follows [24]:

(3) $$U\left(x\right)=-\frac{1}{2}Ax^{2}+\frac{1}{4}Bx^{4}$$

where A and B are the potential parameters. Figure 3 shows the two potential wells of the bi-stable stochastic resonance model. The two minima are located at *x* = ±√(*A*/*B*) and *x* = 0, and the potential depth value is Δ*U* = *A*^2^/(4*B*).

## 3. Principle of Magnetic Anomaly Detection

### 3.1. Compound Tri-Stable Stochastic Resonance Model

**Figure 4:**
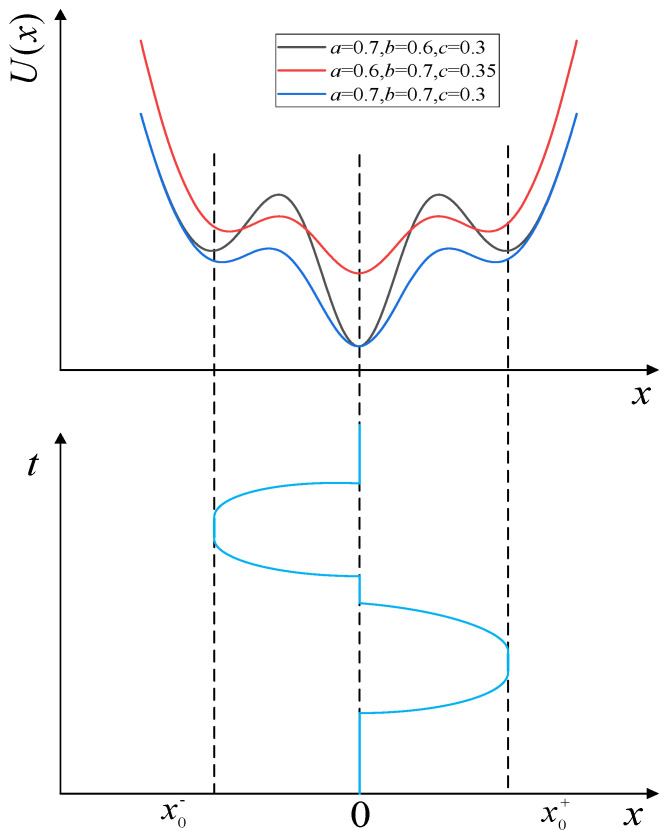
CTSR potential function model and model response.

In terms of the utilization of noise, a TTSR model performs better than a BSR model in the detection of weak signals. However, the parameters of a TTSR model have the problem of coupling, which is not conducive to regulation. To address this problem, a CTSR model is proposed, which can both utilize the noise more effectively and achieve independent regulation of the parameters. The CTSR potential function model is obtained by combining a Gaussian Potential (GP) model with a mixed bi-stable stochastic resonance (MBSR) model. The expressions are as follows:

(4) $$\left\{\begin{array}{l} U\left(x\right)=U_{1}\left(x\right)+U_{2}\left(x\right)\\ U_{1}\left(x\right)=ae^{\left(\left(-x^{2}/b^{2}\right)\right)}\\ U_{2}\left(x\right)=e^{\left(-x^{2}\right)}+\frac{cx^{2}}{2} \end{array}\right.$$

where *U_1_*(*x*) is the GP model, *a* controls the depth of the potential well, *b* controls the width of the potential well, *U_2_*(*x*) is the MBSR model, and *c* controls the barrier height and the steepness of the potential wall. As *c* gradually increases from 0.1 to 0.5, the height of the potential barrier gradually decreases, but the steepness of the potential wall gradually increases. The CTSR potential function has two potential barriers and three potential wells, as shown in Figure 4.

The MBSR model controls the potential wells at both ends, and the potential well in the middle is controlled by the GP model, achieving independent regulation of the potential wells. As *a* decreases from 0.8 to 0.6, the width of the potential wells remains the same, the depth of the intermediate potential well gradually decreases, and the particle consumes less energy to jump to the potential well. As *b* increases from 0.4 to 0.7, the depth of the middle potential well remains constant and the steepness of the potential wells on both sides gradually flattens out, facilitating the particle leap. From Figure 3, it can be seen that the locations of the minima are *x* = 0, *x* = *x*_0_^−^, and *x* = *x*_0_^+^ (where *x*_0_^−^ and *x*_0_^+^ are obtained from Equation (5)), and two potential barriers (maxima) separate the three minima zones.

(5) $$e^{\left(-x^{2}/b^{2}\right)}+\frac{b^{2}}{a}e^{\left(-x^{2}\right)}=\frac{cb^{2}}{2a}$$

The initial state of the CTSR model is at *x* = 0. When there is no magnetic anomaly signal input, the model output is *x*(*t*) = 0. When there is a magnetic signal input, the model output jumps to *x* = *x*_0_^−^ or *x* = *x*_0_^+^, and after the input disappears, the system returns to the initial state at *x*(*t*) = 0 under the action of noise energy.

**Figure 5:**
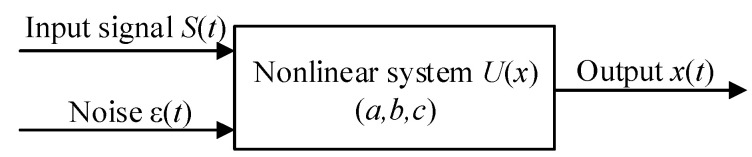
CTSR model input and response.

In the stochastic resonance process, the trajectory of Brownian particles in the potential field is described by the Langevin equation, which is expressed by

(6) $$x\left(t\right)=\frac{dx}{dt}=\frac{dU\left(x\right)}{dt}+S\left(t\right)+\varepsilon \left(t\right)$$

where *S*(*t*) is the magnetic anomaly signal, *ε*(*t*) is the geomagnetic noise signal, and *x_t_* is the output of the CTSR model. The schematic diagram of the working principle of Equation (6) is shown in Figure 5.

### 3.2. CTSR Model for Magnetic Anomaly Detection

From Equations (4) and (6), we obtain

(7) $$\frac{dx}{dt}=-\frac{2ax}{b^{2}}e^{\left(-x^{2}/b^{2}\right)}-2xe^{\left(-x^{2}\right)}+cx+S\left(t\right)+\varepsilon \left(t\right)$$

The actual signal processed by Equation (7) is discrete. The discretized Equation (6) is solved using the Runge–Kutta equation. We obtain

(8) $$\left\{\begin{array}{l} f\left(x\right)=-\frac{2ax}{b^{2}}e^{\left(-x^{2}/b^{2}\right)}-2xe^{\left(-x^{2}\right)}+cx\\ x_{n+1}=x_{n}+\frac{h}{6}\left(k_{1}+2k_{2}+2k_{3}+k_{4}\right)\\ k_{1}=h\left[-\frac{2ax_{n}}{b^{2}}e^{\left(-x_{n}{}^{2}/b^{2}\right)}-2x_{n}e^{\left(-x_{n}{}^{2}\right)}+cx_{n}+u_{n}\right]\\ k_{2}=h\left\{-\frac{2a\left(x_{n}+\frac{k_{1}}{2}\right)}{b^{2}}e^{\left[-{\left(x_{n}+\frac{k_{1}}{2}\right)}^{2}/b^{2}\right]}-2\left(x_{n}+\frac{k_{1}}{2}\right)e^{\left[-{\left(x_{n}+\frac{k_{1}}{2}\right)}^{2}\right]}+c\left(x_{n}+\frac{k_{1}}{2}\right)+u_{n+1}\right\}\\ k_{3}=h\left\{-\frac{2a\left(x_{n}+\frac{k_{2}}{2}\right)}{b^{2}}e^{\left[-{\left(x_{n}+\frac{k_{2}}{2}\right)}^{2}/b^{2}\right]}-2\left(x_{n}+\frac{k_{2}}{2}\right)e^{\left[-{\left(x_{n}+\frac{k_{2}}{2}\right)}^{2}\right]}+c\left(x_{n}+\frac{k_{2}}{2}\right)+u_{n+1}\right\}\\ k_{4}=h\left\{-\frac{2a\left(x_{n}+k_{3}\right)}{b^{2}}e^{\left[-{\left(x_{n}+k_{3}\right)}^{2}/b^{2}\right]}-2\left(x_{n}+k_{3}\right)e^{\left[-{\left(x_{n}+k_{3}\right)}^{2}\right]}+c\left(x_{n}+k_{3}\right)+u_{n+2}\right\} \end{array}\right.$$

where *h* is the time step (sampling interval time), *u_n_* is the mixed signal of magnetic anomaly and noise, and *x_n_* is the output of the CTSR model.

Stochastic resonance requires the interaction of a weak signal, potential function, and noise. The stochastic resonance process works best when the three are optimally matched. The SNR of the CTSR model is established as an indicator to optimize the parameters of the CTSR model, and the SNR is defined as follows:

(9) $$SNR=10\log_{10}\left(\frac{\overset{N}{\underset{i=1}{\sum }}x\left(n\right)}{\overset{N}{\underset{i=1}{\sum }}\varepsilon \left(n\right)}\right)$$

where *N* is the length of the time series, and a higher output SNR indicates that the weaker feature signals are extracted. The optimization range of each parameter is set to [0, 5], and the basic parameters are set as in the quantum genetic algorithm (QGA), including the population size *P*, the length *B* of each binary variable, and the number of final iteration generations *G*.

In the case of simulating three typical magnetic anisotropic signals superimposed with measured noise samples, three synthetic signals *u_n_* are obtained, and the basic parameters are set in the genetic algorithm. The specific operation is as follows.

(1) The range of parameters to be optimized is set to $a,b,c\in \left[0,3\right]$. Set basic parameters in the quantum genetic algorithm are set to *P* = 40, *B* = 20, and *G* = 50. This initialization is set to ensure a high convergence rate and an acceptable calculation time as a rule of thumb.
(2) The maximum output SNR by the QGA and crystal-optimized parameters *a*, *b*, and *c* are found.
(3) The optimized parameters are substituted into the CTSR system to obtain the best output waveform.

### 3.3. Statistical Filtering-Based Judgment System

Without processing, the *x_n_* output of the CTSR model response is used as the basis for the final verdict on the presence or absence of the magnetic anomaly signal, which is prone to false verdicts or false alarms. Therefore, a filter and a judgment system need to be added to the output of the CTSR model to improve the detection probability. We designed a statistical filtering-based judgment system, which performs statistical analysis on data within a certain domain, filters out noisy data that do not meet the conditions, and sets thresholds *d*^−^*_threshold_* and *d*^+^*_threshold_* as judgment thresholds.

**Figure 6:**
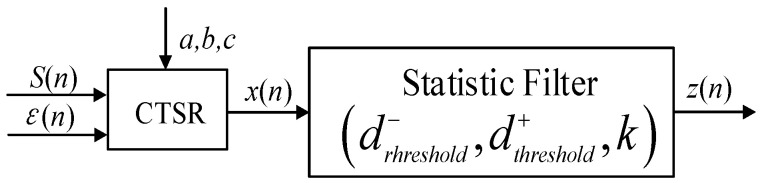
Detection diagram of the CTSR system.

A model for processing the response signal *x_n_* with the statistical filter in Figure 6 is shown in Equations (10) and (11).

(10) $$\left\{\begin{array}{l} \bar{x}=\frac{1}{m}\overset{n}{\underset{i=n-m+1}{\sum }}x\left(i\right)\\ \sigma =\sqrt{\frac{1}{m}\overset{n}{\underset{i=n-m+1}{\sum }}{\left(x\left(i\right)-\bar{x}\right)}^{2}}\\ k=int\left[\max\left(\frac{\left|x\left(i\right)-\bar{x}\right|}{\sigma }\right)\right]+1\\ d_{threshold}^{+}=\bar{x}+k\sigma \\ d_{threshold}^{-}=\bar{x}-k\sigma \end{array}\right.$$

(11) $$\left\{\begin{array}{l} z\left(n\right)=0x\left(i\right)\in \left(d_{threshold}^{-},d_{threshold}^{+}\right)\\ z\left(n\right)=-1x\left(i\right)\in \left(-\infty ,d_{threshold}^{-}\right)\\ z\left(n\right)=1x\left(i\right)\in \left(d_{threshold}^{+},+\infty \right) \end{array}\right.$$

where *m* is the sliding window size, $\bar{x}$ is the mean of the response signal *x_n_* within the sliding window, *σ* is the standard deviation of the response signal *x_n_* within the sliding window, *d*^−^*_threshold_* dthreshold-and *d*^+^*_threshold_* are the standard deviation thresholds, *z*(*n*) is the output of the statistic filter, and *k* is the adaptive standard deviation threshold factor, which is set according to the rule of thumb to ensure that the judgment is more accurate.

## 4. Experiment

**Figure 7:**
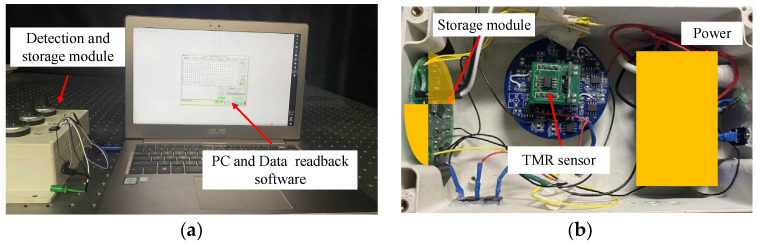
Experimental system: (**a**) data acquisition system; (**b**) internal structure diagram of the detection and storage module.

To obtain realistic geomagnetic noise samples, the experimental test system uses a tunnel magneto-resistance (TMR) sensor (TMR9002) and the corresponding voltage amplifier circuit, self-made storage equipment (the sampling frequency is 2 kHz), and a notebook computer, which runs data readback software and data processing software (as shown in Figure 7).

The TMR9002 sensor uses a push–pull Wheatstone full bridge structure. Its specific parameters are shown in Table 1.

**Figure 8:**
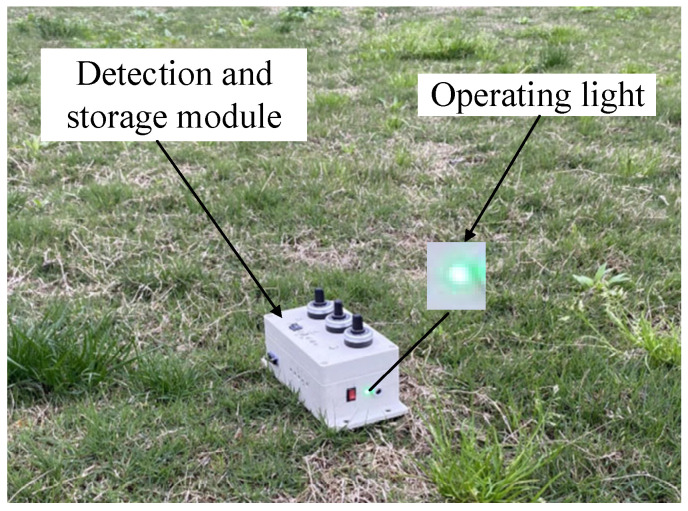
Geomagnetic measurement in an open space.

**Figure 9:**
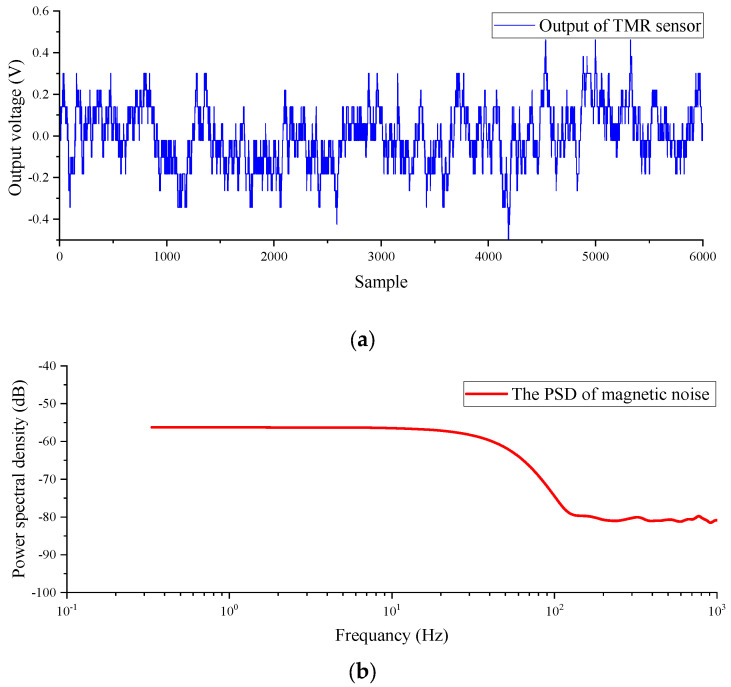
(**a**) Background magnetic field noise; (**b**) background magnetic field power spectral density.

In an open area devoid of magnetic interference, as illustrated in Figure 8, such as public frequency noise, the test system was employed to measure the geomagnetic field. The obtained real geomagnetic noise comprises the actual geomagnetic field along with the intrinsic noise of the TMR sensor and the noise parameters of the amplifier circuit. The true geomagnetic noise and its corresponding power spectral density (PSD) are depicted in Figure 9.

**Figure 10:**
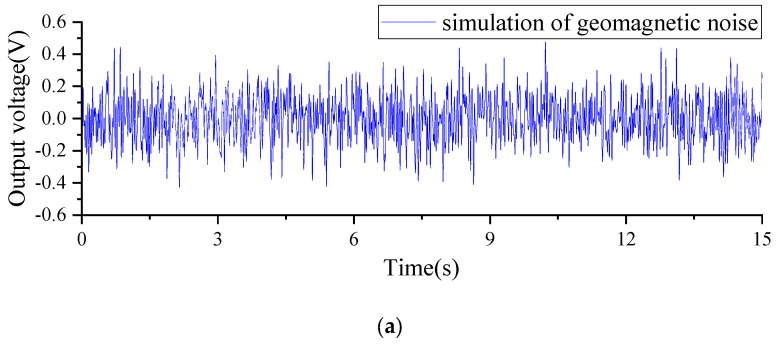

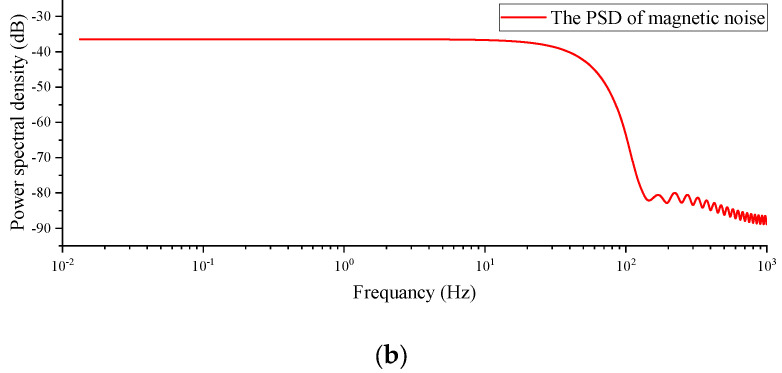
(**a**) Simulated background magnetic field noise; (**b**) simulated background magnetic field power spectral density.

Based on the inherent characteristics of natural geomagnetic noise, we designed a simulated geomagnetic noise signal that closely resembles the power spectral density of the real geomagnetic noise. The simulated geomagnetic noise and its corresponding power spectral density are presented in Figure 10.

**Figure 11:**
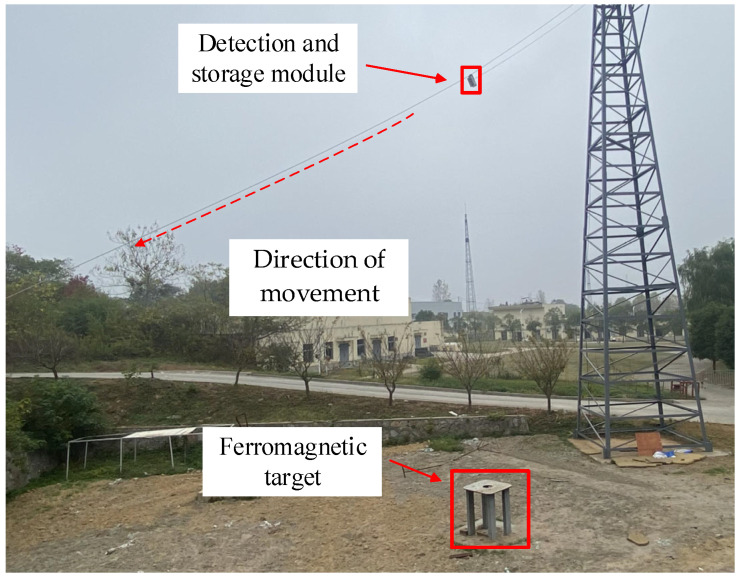
Magnetic anomaly detection test.

Field testing was performed according to the magnetic anomaly detection model in Figure 1, and the test method is shown in Figure 11. The detection and storage module glides from the top of the tower (height of about 15 m) to the ground at a constant speed (4–5 m/s) by motor control. The ferromagnetic target is placed on the ground below the direction of movement. Three different typical magnetic anomaly signals are obtained by adjusting the direction of movement of the detection and storage module and the position of the ferromagnetic target. The ferromagnetic target has a length of 50 cm, a width of 50 cm, and a height of 60 cm.

**Figure 12:**
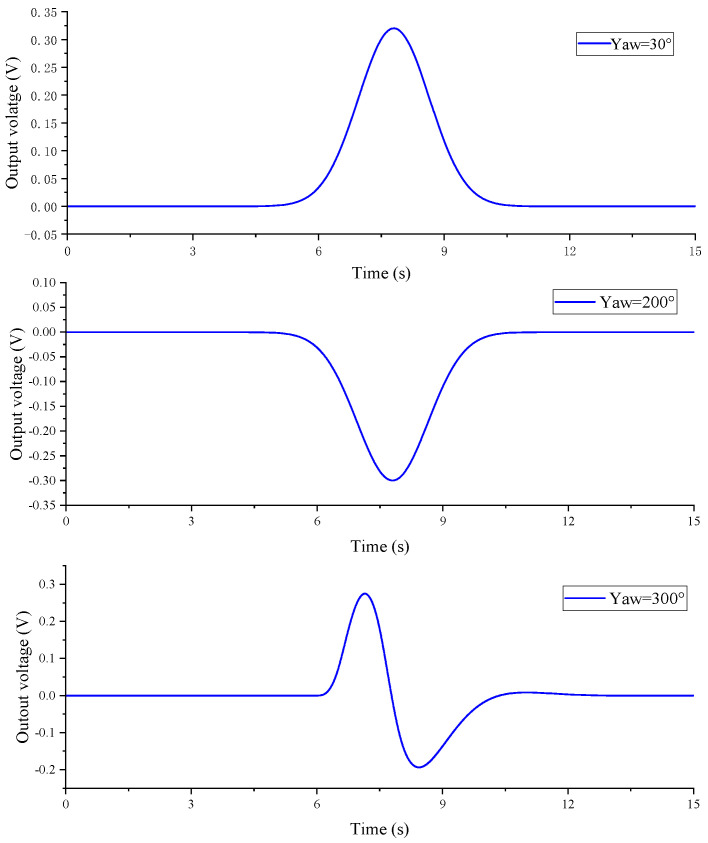
Typical magnetic anomaly signal from the TMR sensor output.

By taking into account the sensitivity of the TMR sensor and the amplification provided by the hardware op amp circuit, we have transformed the three typical magnetic anomaly signals obtained from the simulation into voltage signals outputted by the sensor. These voltage signals are illustrated in Figure 12.

In Figure 12, the typical magnetic anomaly signal obtained from the output of the TMR sensor is combined with the real geomagnetic field noise as the input to the CTSR system. The system parameters, namely a = 0.05, b = 0.2, and c = 0.35, were optimized using QGA and SNR metrics. For more detailed information, please refer to Section 3.2.

**Figure 13:**
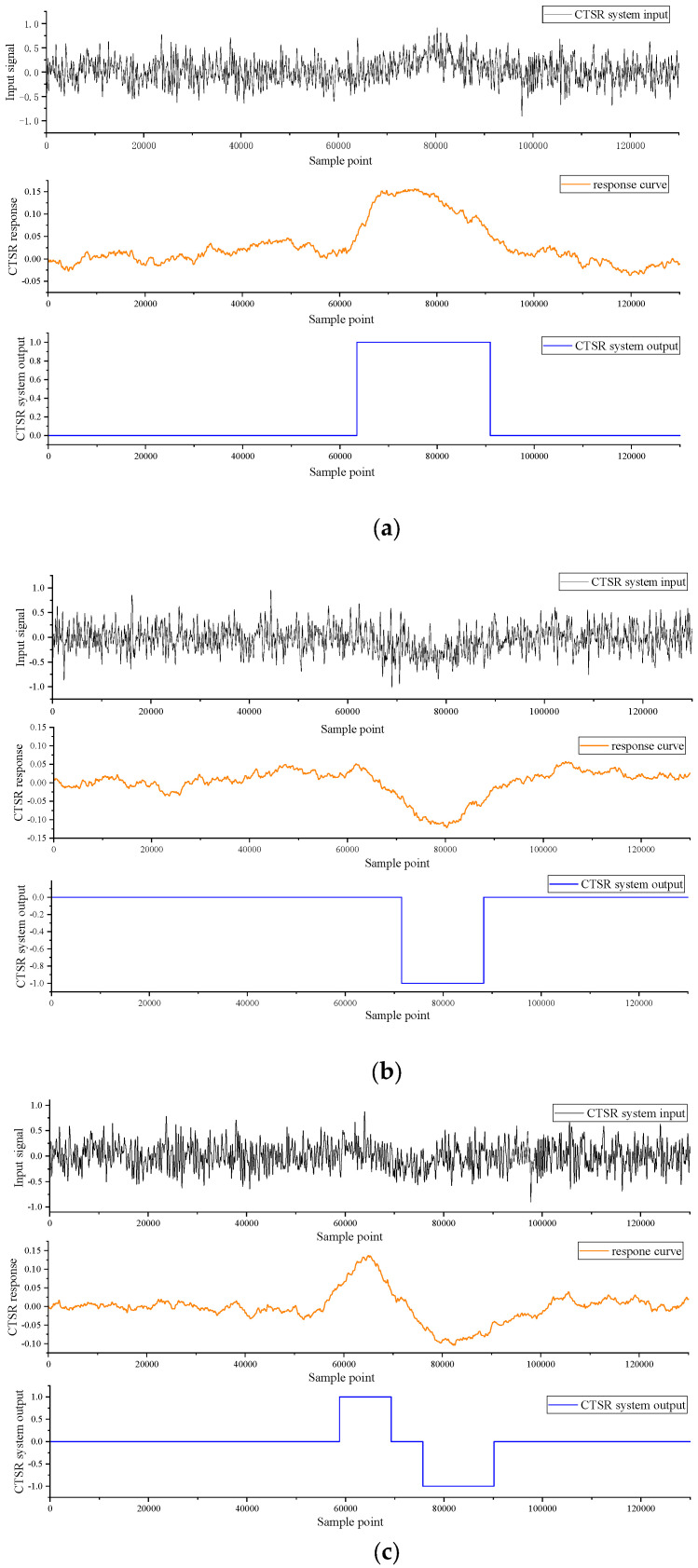
Responses of the CTSR system to the field test. (**a**) The response of the CTSR system to the peak signal; (**b**) The response of the CTSR system to the trough signal; (**c**) The response of the CTSR system to the peak and trough signal.

The responses of the CTSR system to the field test are shown in Figure 13. Where (a) is the mixed signal of the magnetic anomaly of the sensor with the simulated geomagnetic noise, the response output of the composite tri-stable stochastic resonance detection peak signal, and the verdict signal of the CTSR system; (b) is the mixed signal of the sensor where the magnetic anomaly with the geomagnetic noise, the response output of the composite tri-stable stochastic resonance detection trough signal, and the judgment signal of the CTSR system; and (c) is the mixed signal of the sensor moving magnetic anomaly with the geomagnetic noise, the response output of the composite tri-stable stochastic resonance detection peak and trough signal, and the judgment signal of the CTSR system.

**Figure 14:**
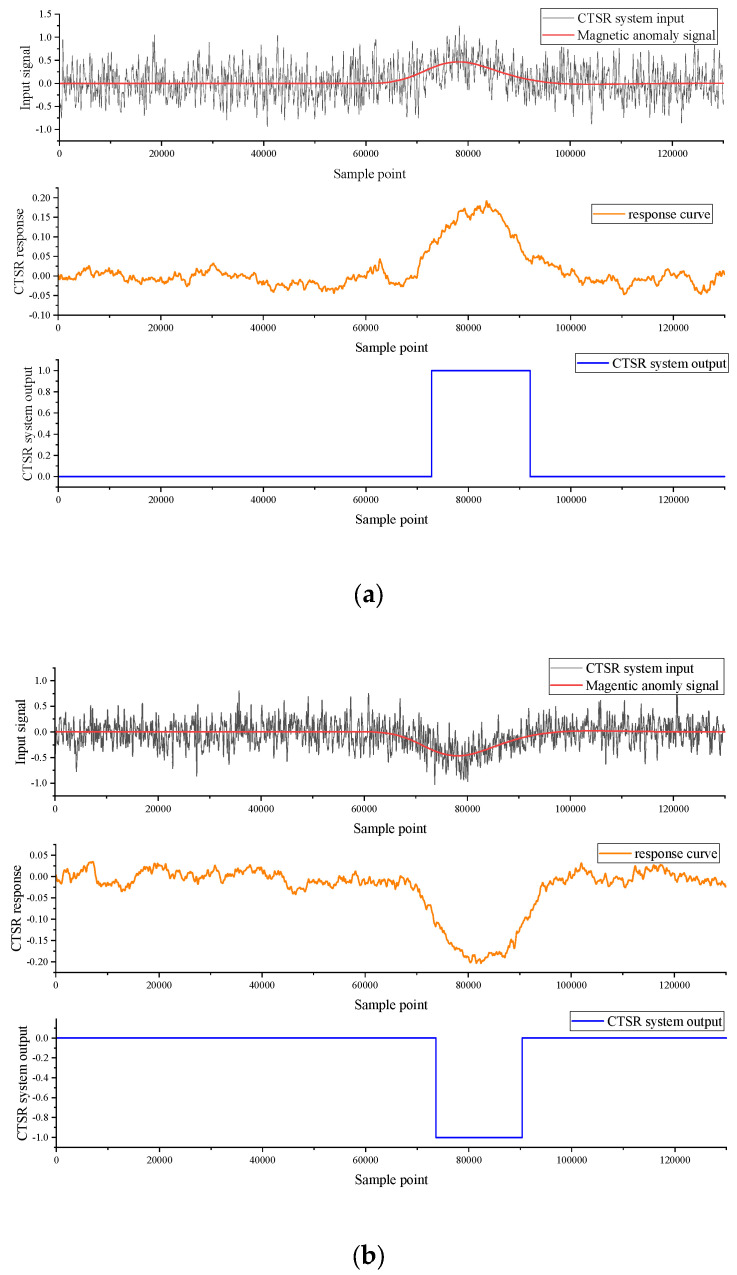

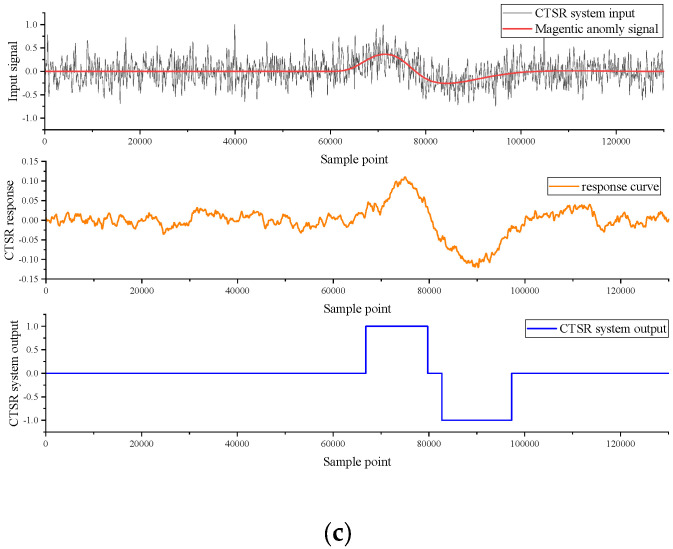
CTSR system response diagram (**a**) Yaw = 30°; (**b**) Yaw = 200°; (**c**) Yaw = 300°.

The CTSR system response to two synthetic input signals is shown in Figure 14, (SNR = −5.5 dB), where (a) is the mixed signal of the magnetic anomaly of the sensor moving along a 30° angle with the simulated geomagnetic noise, the response output of the composite tri-stable stochastic resonance detection peak signal, and the verdict signal of the CTSR system; (b) is the mixed signal of the sensor where the magnetic anomaly moves along the 200° direction with the simulated geomagnetic noise, the response output of the composite tri-stable stochastic resonance detection trough signal, and the judgment signal of the CTSR system; and (c) is the mixed signal of the sensor moving magnetic anomaly along the 300° direction with the simulated geomagnetic noise, the response output of the composite tri-stable stochastic resonance detection peak and trough signal, and the judgment signal of the CTSR system.

**Figure 15:**
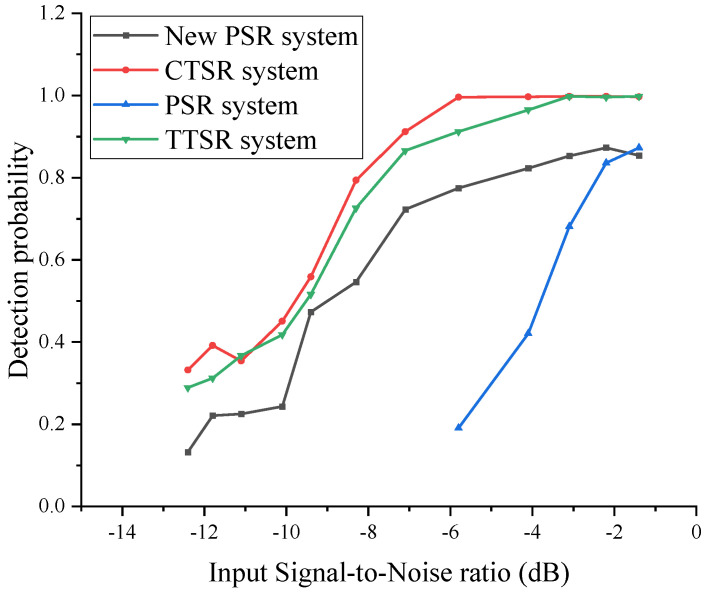
Detection system monitoring a probability comparison chart.

To assess the performance of the CTSR system, both the CTSR and the PSR detection effects in [22] were compared. The Monte Carlo method was used to calculate the detection probability of magnetically dissimilar signals at different SNRs. We added randomly generated simulated geomagnetic field noise to the typical magnetic anomaly signal output from the TMR sensor and calculated the detection probability of the magnetic anomaly signal under different SNR conditions. Each detection probability is a statistical value of the results of 10,000 repeated detections. We added the new PSR system for comparison, where the judgment method is replaced with a statistical filtering-based judgment system. The monitoring probabilities of the three magnetic anomaly detection systems at different SNRs are shown in Figure 15.

The CTSR and new PSR systems basically maintain a stable, high monitoring probability at SNRs greater than −6 dB, and the detection probabilities of both detection systems decline significantly at SNRs less than −6 dB. When the input SNR is −1 dB, the detection effect of the PSR detection system is similar to that of the new PSR detection system. When the input SNR is between −1 and −6 dB, the monitoring probability of the PSR detection system drops sharply. The CTSR detection system performs significantly better than the new PSR and other detection systems, and the detection probability is close to 100% at SNRs less than −6 dB. CTSR offers the advantage of independently adjusting the depth and width of the potential well compared to TTSR. Therefore, it is evident from Figure 15 and Table 2 that CTSR exhibits a more pronounced effect of stochastic resonance under low SNR conditions.

## 5. Discussions

(1) This study uses randomly generated simulated geomagnetic noise in order to demonstrate the detection capability of the CTSR system. The number of real geomagnetic noise samples collected is limited. The simulated geomagnetic noise power spectral density basically conforms to a distribution law of 1/*f ^α^* (0 < *α* < 2) and is similar to the real geomagnetic noise power spectral density distribution.

(2) The judgment system in the new PSR system proposed in this paper is the proposed statistical filtering-based judgment system. The output of the stochastic resonance model can identify magnetic signals without interpotential jumps, but it leads to false alarms in the detection results. The monitoring probability is effectively improved compared to the detection results in [22].

(3) The input to the CTSR system is a superposition of three random typical magnetic anomaly signals and no magnetic anomaly signals with randomly generated simulated geomagnetic field noise. Only if the output is 1 or −1 within the time point (*n* = 60,000~100,000) when the magnetic anomaly signal appears is it a valid detection; if no magnetic anomaly signal and the CTSR system outputs are 1 or −1, it is a false alarm.

(4) In this study, *n* = 20,000 and *m* = 20,000 in Equation (10) of the statistical filtering-based judgment system. The first 20,000 points are the stochastic resonance system output with geomagnetic noise. The system can calculate the adaptive standard deviation threshold factor *k* to effectively improve the detection probability of the CTSR system.

(5) The CTSR system improves the stability of magnetic anomaly signal detection and preserves the relative motion (heading) information between the ferromagnetic target and the detection module.

(6) The minimum magnetic field detection ability of the magnetic sensor is related to its sensitivity and background noise. The background noise power spectral density conforms to a distribution law of 1/*f ^α^* (0 < *α <* 2). Low frequency 1/*f ^α^* noise is ubiquitous in various electronic devices. Due to the difference in sensitivity and background noise of the magnetic sensor, the energy barrier of the stochastic resonance system of the CTSR system is different. Finally, the system parameters (*a*, *b*, and *c*) are changed.

(7) The conclusion of this article is the result obtained by Monte Carlo simulation. This method is difficult to implement if a large number of tests are repeated. In the future, our research will focus on designing a more efficient platform for the intersection of target and detector. Based on this platform, more experimental data in the case of intersection will be supplemented in future studies.

## 6. Conclusions

The CTSR model has a higher noise utilization rate. The individual potential wells of the CTSR model are independently regulated by the system parameters, and there is no system parameter coupling. The monitoring probability is close to 80% when the input SNR is −8 dB, and the detection probability is close to 100% at SNRs greater than −6 dB. The experimental results show that the CTSR system can effectively detect and extract magnetic anomaly signals in the absence of a priori information and low SNRs. Compared to PSR systems, the CTSR system is simpler in design, more resistant to interference, and has a much higher noise utilization rate. In the case of limited conditions of the magnetic detection device, there is great potential to develop a scheme to improve the detection distance of the detection device.

## Figures and Tables

**Table 1:** Features of TMR9002.

Saturated Field	Sensitivity	Background Noise
±8Oe	100 mV/V/Oe	<150pT/rt(Hz)@1 Hz

**Table 2:** Detection systems that detect the probability of magnetic anomaly signals.

Detector	Detection Probability	
	SNR = −8 dB	SNR = −6 dB
PSR system	<0.01	0.191
New PSR system	0.546	0.775
TTSR system	0.726	0.912
CTSR system	0.794	0.996

## Data Availability

The data presented in this study are available on request from the corresponding author. The data are not publicly available due to privacy.
